# Task-Related EEG as a Biomarker for Preclinical Alzheimer’s Disease: An Explainable Deep Learning Approach

**DOI:** 10.3390/biomimetics10070468

**Published:** 2025-07-16

**Authors:** Ziyang Li, Hong Wang, Lei Li

**Affiliations:** Department of Mechanical Engineering and Automation, Northeastern University, Wenhua Street, Shenyang 110819, China

**Keywords:** task-related EEG, Alzheimer’s disease risk, interpretable deep learning, early screening, biomarker

## Abstract

The early detection of Alzheimer’s disease (AD) in cognitively healthy individuals remains a major preclinical challenge. EEG is a promising tool that has shown effectiveness in detecting AD risk. Task-related EEG has been rarely used in Alzheimer’s disease research, as most studies have focused on resting-state EEG. An interpretable deep learning framework—Interpretable Convolutional Neural Network (InterpretableCNN)—was utilized to identify AD-related EEG features. EEG data were recorded during three cognitive task conditions, and samples were labeled based on APOE genotype and polygenic risk scores. A 100-fold leave-p%-subjects-out cross-validation (LPSO-CV) was used to evaluate model performance and generalizability. The model achieved an ROC AUC of 60.84% across the tasks and subjects, with a Kappa value of 0.22, indicating fair agreement. Interpretation revealed a consistent focus on theta and alpha activity in the parietal and temporal regions—areas commonly associated with AD pathology. Task-related EEG combined with interpretable deep learning can reveal early AD risk signatures in healthy individuals. InterpretableCNN enhances transparency in feature identification, offering a valuable tool for preclinical screening.

## 1. Introduction

Alzheimer’s disease (AD) is a progressive neurodegenerative disorder and the leading cause of dementia worldwide [1]. The disease imposes a significant burden on individuals and healthcare systems due to its insidious onset, its irreversible progression, and the current lack of curative treatments. A critical challenge in clinical practice is the early and accurate identification of individuals at risk of AD, ideally before the onset of irreversible cognitive decline [2]. However, conventional diagnostic methods, such as neuroimaging and cerebrospinal fluid biomarkers, are expensive, invasive, or inaccessible in many settings, limiting their scalability for population-level screening [3].

Mild cognitive impairment (MCI), especially its amnestic subtype, is widely recognized as an intermediate stage between normal aging and AD [4]. The early identification of MCI or even cognitively normal individuals at elevated risk is essential for timely intervention and disease prevention [5]. Studies suggest that approximately 10–15% of individuals with MCI progress to AD annually [6], underscoring the importance of accurate risk stratification tools that can support early detection strategies. In the strict medical definition, MCI is considered part of the clinical stage of AD, as it involves detectable cognitive symptoms that can be observed, evaluated, and diagnosed [7]. In contrast, the preclinical stage refers to a phase during which pathological changes—such as amyloid plaques and tau tangles—begin to accumulate in the brain [8], but individuals do not yet exhibit any subjective complaints or objective signs of cognitive impairment [9]. Detecting individuals in this asymptomatic preclinical phase is critical for early intervention, as neuronal damage may already be progressing silently [10].

Recent research efforts have thus shifted toward identifying biomarkers that can capture these subtle neurobiological changes before clinical symptoms emerge [11,12]. Electroencephalography (EEG), with its high temporal resolution and sensitivity to early functional brain alterations, as a non-invasive, low-cost, and portable modality [13,14], offers a promising avenue for large-scale early AD screening [15,16,17]. EEG has demonstrated sensitivity to early brain changes associated with AD pathology, such as increases in theta activity, reductions in alpha power, and disruptions in slow-wave dynamics [18,19,20]. While most prior EEG studies have focused on MCI or dementia stages, emerging work suggests that even cognitively healthy individuals carrying genetic risk alleles (e.g., APOE ε4) may show distinctive electrophysiological patterns [21], particularly during task-related cognitive engagement [22]. Dzianok and Kublik [23] found that Alzheimer’s disease risk alleles of the PICALM gene may accelerate cognitive functions, such as memory and attention, in cognitively normal, middle-aged individuals carrying the APOE ε4 allele. Longitudinal studies have further shown that EEG-derived features correlate with biomarkers of amyloid-beta deposition and neurodegeneration. For example, a five-year follow-up study involving 304 older adults reported predictive accuracies of 62% for EEG and 61% for MRI in detecting AD-related pathological changes [24]. Despite these advances, the clinical utility of EEG-based models remains limited due to insufficient interpretability.

Remarkably, the field has witnessed growing interest in explainable deep learning methods [25,26,27], which aim to reveal how models make predictions and which features contribute most significantly [28,29]. This is particularly important in medical applications, where understanding the decision process is critical for clinical trust and regulatory acceptance [30]. Techniques such as attention mechanisms, saliency mapping, and layer-wise relevance propagation have been introduced to uncover spatial–temporal EEG signatures [31,32,33] relevant to various cognitive tasks and neurological conditions. Khosravi et al. [34] proposed CL-ATBiLSTM to capture temporal dependencies within EEG sequences. Cui et al. [35] proposed the InterpretableCNN model to detect drowsiness, and they introduced a novel interpretability technique based on class activation maps (CAMs) [36,37], which reveals localized input regions critical to the model’s predictions. The InterpretableCNN model is more straightforward and directly captures common patterns associated with different mental states from raw EEG signals, without the need for the manual extraction of time–frequency features.

In this study, we introduce an interpretable EEG-based framework for identifying AD-related risk in cognitively healthy individuals carrying genetic risk markers (specifically, APOE ε4 allele carriers). By integrating neural attention mechanisms and a frequency-domain analysis, we aim to uncover distinct spatial–temporal patterns of brain activity associated with increased AD risk. To the best of our knowledge, this is the first attempt to use explainable models to detect subtle task-related EEG alterations in asymptomatic individuals at genetic risk of AD, offering new insights into preclinical biomarkers and their potential applications in precision prevention. The main aims and contributions of this paper are as follows:

- This study introduces an interpretable framework for identifying task-related EEG features indicative of Alzheimer’s disease risk, providing insights into the underlying neural mechanisms.
- Theta and alpha oscillatory features in the parietal and temporal regions are identified as key biomarkers for early AD risk, aligned with established neurodegenerative patterns.
- This study highlights the potential of developing cost-effective assessment tools and biomarkers that are highly sensitive to cognitive decline and neurological dysfunction in clinically healthy populations at the preclinical stage.

## 2. Materials and Methods

### 2.1. Data Preparation

The dataset utilized in this study is publicly accessible [38] and originates from research exploring the association between brain structure/function and fundamental health metrics in cognitively normal, middle-aged individuals carrying genetic risk factors. Participants were categorized based on the presence of two known risk-related genes: APOE (apolipoprotein E) and PICALM (phosphatidylinositol binding clathrin assembly protein). The classification comprised three groups: non-carriers (N), individuals carrying only the APOE risk variant (A+P−), and those carrying risk variants in both APOE and PICALM (A+P+) [21]. For each group, data collection included EEG recordings under both resting-state and task-based conditions, specifically during the Sternberg Task (STMT) and Multi-Source Interference Task (MSIT), as well as functional MRI (fMRI) scans.

**Figure 1:**
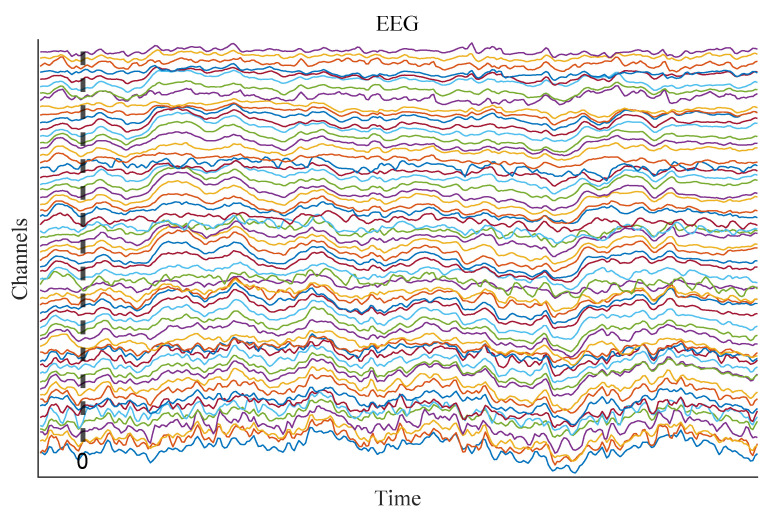
Preprocessed sample of raw signals that were used for deep learning.

The original EEG recordings were obtained using a Brain Products system equipped with 128 electrodes. During acquisition, FCz served as the online reference, and signals were sampled at a rate of 1000 Hz. To ensure data integrity and signal quality, electrode impedances were kept within the range of 5 to 10 kΩ throughout the recording process. Following the preprocessing of the EEG signals and the exclusion of invalid trials, a total of 51 participants were retained for analysis (for comprehensive methodological details, please refer to our earlier work [22]). Offline EEG signals were re-referenced to approximate the average mastoid. Groupwise demographic characteristics of the participants and corresponding sample sizes under different conditions are summarized in Table 1. Each trial yielded a data segment comprising 60 EEG channels and 320 time points (sampling rate: 250 Hz), spanning a time window from −80 to 1200 ms relative to stimulus onset, as shown in Figure 1.

### 2.2. InterpretableCNN

We adopted the Interpretable Convolutional Neural Network (InterpretableCNN) architecture proposed by Jian Cui et al. (2023) [35], which has shown strong capability in extracting biologically meaningful features from EEG signals. In this study, the model was applied directly to the preprocessed data, with necessary adjustments to the input and output dimensions to align with the experimental setup, as summarized in Table 2. The network architecture comprises seven layers, as illustrated in Table 2. The initial two layers employ pointwise and depthwise convolution operations. These are subsequently followed by a ReLU activation function, a batch normalization layer, a global average pooling layer, a fully connected (dense) layer, and a final Softmax activation layer for classification.

In a typical separable convolution [39], the depthwise operation extracts spatial features independently for each input channel, followed by a pointwise (1 × 1) convolution that integrates inter-channel information and projects the result into a new feature space. However, this model uses the pointwise convolution to implement the first processing step (1) of demixing the signals and the depthwise convolution to implement the second processing step (2) of learning temporal features from each demixed signal independently. In addition, interpretation techniques inspired by the CAM and Fixation-CNN methods were developed to visualize the model’s decision-making process by highlighting the input signal components that contributed most significantly to the classification [35]. This means that InterpretableCNN can reveal the important local regions of the sample for the classification.

### 2.3. Implementation Details

Due to the limited number of valid subjects in the A+P+ group, which precluded matching for age and gender distributions across groups, and to maintain alignment with a single-risk-gene modeling strategy for simulating early-stage risk prediction in a healthy population, only the N and A+P− groups were included in the model training and validation using the leave-p%-subjects-out cross-validation (LPSO-CV) protocol. In accordance with prior implementations of LPSO-CV, we conducted 100 independent iterations of the experiment, each initialized with a different random seed from the range of 42 to 141 to ensure reproducibility [22]. To comprehensively assess model performance during LPSO-CV, we employed several evaluation metrics, including the area under the receiver operating characteristic curve (ROC AUC), Cohen’s Kappa coefficient for agreement assessment, and sensitivity to measure the true-positive detection capability. While earlier studies restricted modeling to N and A+P− groups, the present work incorporates the A+P+ group as an independent high-risk set for post hoc prediction. The sensitivity metric was employed to evaluate model generalizability and to compare its predictive performance against the A+P− subset within the cross-validation framework.

Model training was performed using the Adam optimization algorithm, with a maximum of 200 training epochs. An early stopping criterion was applied, halting training if validation binary accuracy did not improve over 20 consecutive epochs. The training objective was defined by the negative log-likelihood loss (NLLLoss). The training configuration included a learning rate of 0.001 and a batch size of 64. The model implementations were developed in Python 3.8 utilizing the PyTorch 2.2 deep learning framework. Computations were accelerated using an NVIDIA GeForce RTX 4060 GPU, operating within a Ubuntu 20.04 Linux environment.

## 3. Results

### 3.1. Classification Performance

**Figure 2:**
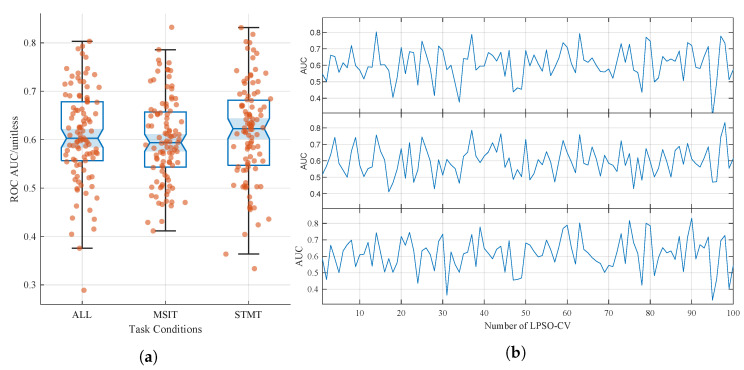
ROC AUC results of LPSO-CV. (**a**) Comparing the performance metrics of ROC AUC scores under three task conditions with significant levels. (**b**) ROC AUC curves across 100 iterations of LPSO-CV. The order of curves from top to bottom in (**b**) corresponds to the task conditions from left to right in (**a**).

As previously described, model performance on the validation set during LPSO-CV was assessed using the ROC AUC, Cohen’s Kappa coefficient, and sensitivity. In addition, sensitivity was employed to evaluate the model’s generalization capability on the A+P+ group, which served as the test set. Figure 2 illustrates the ROC AUC results on the validation set across 100 iterations of LPSO-CV. Figure 2a displays the distribution of the ROC AUC values under the three task conditions. As there were no statistically significant differences based on *t*-tests, no significance markers are included in the figure. Figure 2b presents the ROC AUC curves across 100 iterations of cross-validation for each task condition. The results showed a high degree of consistency among the three conditions. Notably, classification performance varied depending on subject groupings in specific cross-validation folds. For instance, relatively high performance was observed in certain iterations, whereas markedly lower performance occurred in others, such as in the 95th iteration (SEED = 136). These findings suggest that the model is capable of performing cross-subject and cross-task classification, indicating its ability to learn EEG features associated with Alzheimer’s disease risk.

As shown in Table 3, the validation set achieved the highest average ROC AUC under the STMT task condition, reaching approximately 61.44%. The corresponding Cohen’s Kappa coefficient was around 0.23, indicating a fair agreement level of classification performance [40], as well as suggesting that the model was able to effectively capture EEG features associated with genetic risk. Although the classification performance under the MSIT task condition was slightly lower, the average ROC AUC remained close to 60%, and no statistically significant differences were observed compared to the other task conditions. The model trained on the combined dataset across all task conditions achieved moderate classification performance, with an average ROC AUC of 60.84% and a Cohen’s Kappa coefficient of approximately 0.22. These results indicate that the model was able to learn subtle risk-related EEG features even in this highly challenging context of predicting genetic risk in cognitively healthy individuals, yielding a fair agreement in classification.

To assess generalizability, the trained model was used to predict outcomes for the A+P+ group after each cross-validation iteration, and the resulting sensitivity was compared to that of the A+P− samples in the validation set. As shown in Table 3, across all three task conditions, the sensitivity for correctly identifying risk in the A+P+ group was slightly lower than that observed in the A+P− subgroup of the validation set. This outcome is reasonable and supports the model’s capacity to predict AD risk to some extent. Although individuals carrying both risk genes (A+P+) are generally considered to have a higher AD risk than those with a single risk gene (A+P−), the A+P+ group was not involved in model training. Therefore, a slight reduction in sensitivity was expected. This result also reflects an implicit assumption that EEG alterations caused by dual-gene risk may share certain similarities with, yet still differ from, those associated with single-gene risk.

### 3.2. Interpretation of the Learned Characteristics

**Figure 3:**
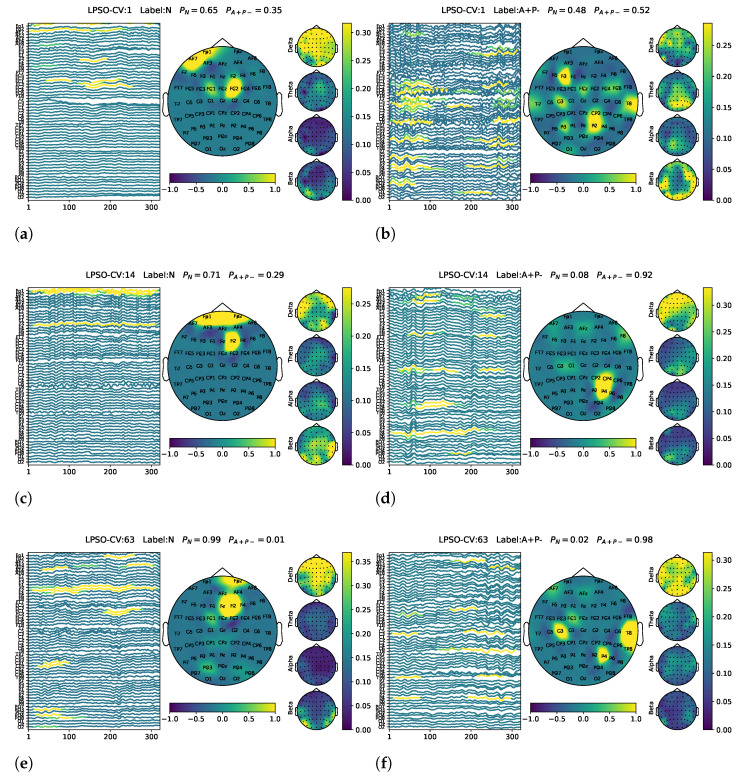
Representative EEG samples selected from multiple LPSO-CV iterations for model interpretability analysis. Left column: EEG trials confidently classified as non-risk gene carriers (N). (**a**) LPSO-CV1, (**c**) LPSO-CV14, (**e**) LPSO-CV63. Right column: EEG trials confidently classified as single-risk gene carriers (A+P−). (**b**) LPSO-CV1, (**d**) LPSO-CV14, (**f**) LPSO-CV63.

Gaining insights into what the model has learned from the data is a crucial aspect of model validation. Figure 3 illustrates representative EEG samples obtained from different LPSO-CV iterations. Compared to the EEG samples that were confidently classified as non-risk gene carriers (N), those confidently identified as single-risk gene carriers (A+P−) generally exhibited more prominent θ or α band activity. Additionally, we observed an interesting spatial pattern: posterior channels located in the parietal and temporal regions, such as T8, P2, and P4, tended to play a more important role in classifying A+P− samples. In contrast, samples classified as non-risk carriers often showed greater reliance on frontal channels, including Fp1, Fp2, F2, and FC2, as illustrated in Figure 3.

**Figure 4:**
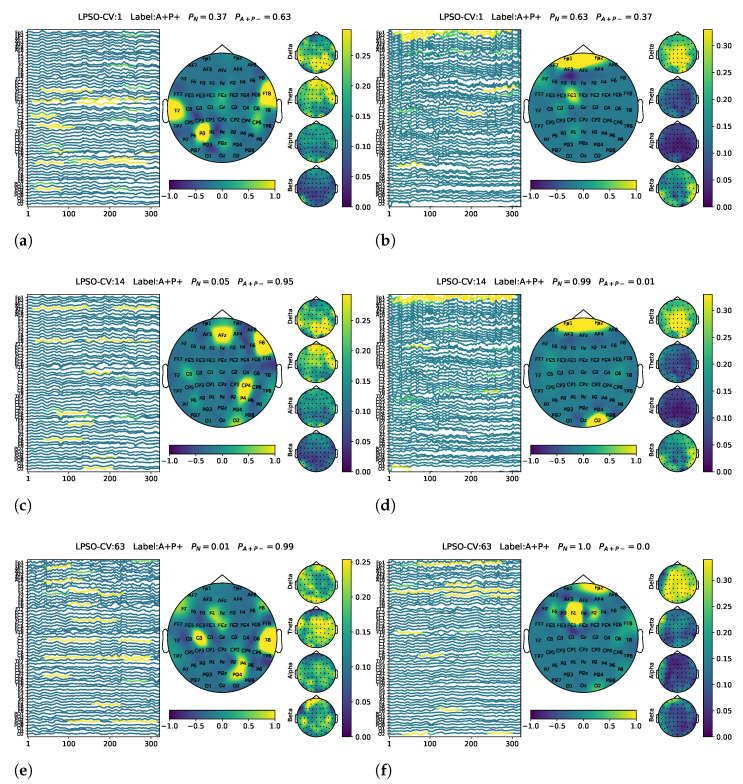
Representative visualization results for generalization to the A+P+ group. Left column: A+P+ samples predicted as A+P−. (**a**) LPSO-CV1, (**c**) LPSO-CV14, (**e**) LPSO-CV63. Right column: A+P+ samples classified as non-risk (N). (**b**) LPSO-CV1, (**d**) LPSO-CV14, (**f**) LPSO-CV63.

Representative visualization results of the model’s generalization to the A+P+ group are presented in Figure 4. The left column shows samples predicted as A+P−, where the model focused on θ and α band activation, with prominent energy in the parietal and temporal regions. This pattern closely resembles that observed in the correctly classified A+P− samples from the validation set (see Figure 3), suggesting that the model demonstrates reasonable generalization when applied to the unseen A+P+ group. The right column shows the A+P+ samples that were misclassified as non-risk (N). These cases typically lack distinct θ and α activation and show weak features in the parietal and temporal channels, which likely contributed to their incorrect classification.

**Figure 5:**
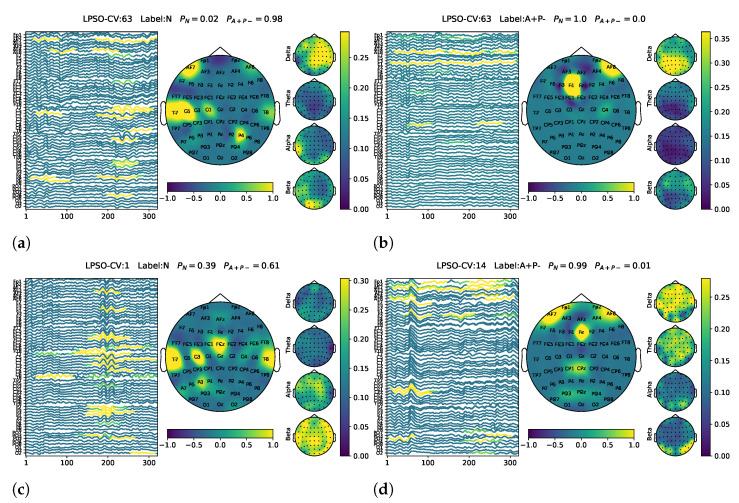
Representative visualization results illustrating misclassification cases. Left column: samples from non-risk carriers (N) misclassified as A+P−. (**a**) Label mismatch feature, (**c**) Electromyographic noise. Right column: A+P− samples misclassified as non-risk carriers (N). (**b**) Label mismatch feature, (**d**) Electroocular noise.

To further investigate the causes of misclassification, the proposed visualization technique was applied to representative samples, as shown in Figure 5. The left column illustrates non-risk carrier samples that were incorrectly classified as A+P−. In some cases, the model captured A+P−-related features, such as alpha band activation in parietal channels (see Figure 5c), which may reflect labeling inaccuracies. Additionally, temporal channels are often contaminated by peripheral muscle activity, potentially misleading the model into detecting spurious risk-related patterns. Even when prefrontal features typical of non-risk carriers were present (see Figure 5a), they were misinterpreted.

The right column shows the A+P− samples that were misclassified as non-risk carriers. Some lacked clear A+P−-related features (see Figure 5b) while exhibiting typical non-risk carrier characteristics. Another contributing factor might be ocular artifacts in the prefrontal region, which are essentially a type of electromyographic noise. Even when parietal theta activity—associated with risk carriers—was present, it was overwhelmed by stronger noise from the frontal area, leading the model to misclassify these samples as non-risk carriers, as shown in Figure 5d.

### 3.3. Unclear and Unexplained Results

The deep learning approach adopted in this study is fundamentally data-driven, aiming to learn AD risk-related biological rhythms and neural activity patterns from EEG signals. Unlike model-driven approaches, which are guided by predefined physiological or theoretical assumptions, data-driven methods rely heavily on the quality of the dataset—particularly the representativeness of the samples and the reliability of the labels. Consequently, if the dataset contains noise, biases, or ambiguous labels, data-driven models may capture spurious correlations or generate misleading patterns that do not generalize well beyond the training data [41,42]. Such reliance on data quality highlights a key limitation of purely data-driven frameworks in clinical neuroscience: while powerful in uncovering hidden patterns, they are also prone to overfitting or generating biologically uninterpretable results when domain-specific constraints are not incorporated [43,44].

**Figure 6:**
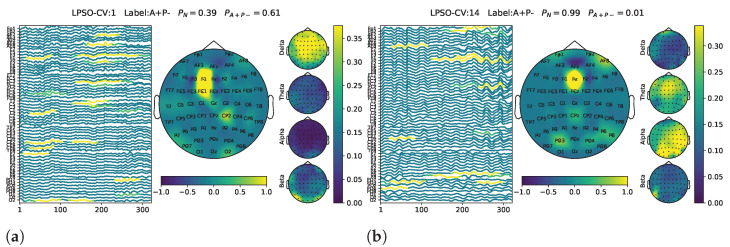
Representative visualization results illustrating unclear cases. (**a**) is closer to N but was classified as A+P−; (**b**) is closer to A+P− but was classified as N.

The whole-band brain topography maps of the two cases shown in Figure 6 appear highly similar. However, substantial differences emerge when examining the frequency-specific topographies. Case (a) exhibits dominant neural activity in the δ band, whereas case (b) shows activity concentrated in the α and β bands. Based on the interpretation provided earlier, case (a) is closer to the N group but was misclassified as A+P−, while case (b) is more similar to the A+P− group but was classified as N. These unclear and unexplained results are most likely attributed to the data-driven nature of the deep learning model, which may learn patterns that are sensitive to subtle data variations rather than robust physiological features. This highlights the potential limitations of purely data-driven approaches in clinical classification tasks, especially in the presence of ambiguous or borderline cases [45]. Clearly, an excessive number of unclear cases leads to insufficient model recognition performance.

## 4. Discussion

In this study, we proposed a deep learning framework to classify cognitively healthy individuals with a genetic risk of Alzheimer’s disease (AD) based on task-related EEG data. The model was evaluated under three cognitive task conditions: MSIT, STMT, and a combined condition. Across 100 repetitions of leave-pair-subjects-out cross-validation (LPSO-CV), the best classification performance was achieved in the STMT task, with an average ROC AUC of 61.44% and a Cohen’s Kappa coefficient of approximately 0.23, reflecting a fair level of agreement [40]. Although the MSIT task yielded slightly lower AUC values, no statistically significant differences were observed among the task conditions, indicating stable model performance. The combined-task setting achieved moderate performance (ROC AUC = 60.84%; Kappa ≈ 0.22), demonstrating the model’s ability to integrate features across heterogeneous cognitive demands. Compared to traditional handcrafted feature approaches, the proposed model offers notable advantages. In our prior work [22], features extracted from time–frequency attention and phase-based spatial–temporal patterns yielded ROC AUCs of 56.98% and 53.72%, respectively, under matched cross-validation protocols. The improvements observed in the current study support the hypothesis that deep learning can better capture complex and subtle EEG representations associated with AD genetic risk, particularly in healthy populations where disease signals may be weak or diffuse. Longitudinal monitoring is critical for identifying reliable biomarkers that predict AD progression. A previous 5-year study involving 304 older adults reported EEG and MRI prediction accuracies of 62% and 61% for amyloid pathology and neurodegeneration, respectively [24]. This further highlights the challenge of identifying AD risk in the preclinical stage, and it underscores the importance of exploring interpretable, non-invasive biomarkers like task-related EEG. Our results preliminarily achieve comparable performance. Kavcic et al. [46] compared the rsEEG spectral density before and after a cognitive task. Although a cognitive task was involved, the analysis focused on resting-state EEG recorded pre- and post-task rather than on task-related EEG signals. To the best of our knowledge, this is the first study to apply task-related EEG-based InterpretableCNN models to predict AD risk in cognitively normal individuals with this level of accuracy.

To assess generalizability, the trained model was applied to an unseen test set (A+P+ group). The sensitivity for risk identification in this group was consistently lower than in the A+P− subgroup from the validation set, which was expected given the absence of these samples during training. The sensitivity was lower in the A+P+ group, indicating that the model may have a limited ability to detect individuals carrying both risk genes. This could be due to the small sample size or the complexity of the genetic risks. Nonetheless, the model was able to detect meaningful theta and alpha band activity in posterior brain regions, such as the parietal and temporal lobes, resembling the patterns found in correctly classified A+P− samples. This suggests that the model learned biologically relevant and generalizable EEG markers of AD risk. Research has shown that memory-related neural circuits (hippocampal–cortical corticothalamic circuits) generate oscillatory events during sleep EEG, including θ bursts (TBs), sleep spindles (SPs), and slow waves (SWs). Alterations in the coupling of these events may indicate early pathophysiological mechanisms underlying AD [47]. Interestingly, similar effects were observed in this study using task-state EEG, suggesting that alterations in oscillatory dynamics associated with memory-related neural circuits may also be detectable outside of sleep.

An interpretability analysis further confirmed the model’s ability to extract neurophysiologically plausible features. Visualizations revealed that high-confidence A+P− predictions were often characterized by stronger theta or alpha band activation in posterior regions, including electrodes such as T8, P2, and P4. In contrast, samples predicted as non-risk (N) typically exhibited frontal activity, particularly in electrodes such as Fp1 and FC2. These observations are consistent with the literature suggesting posterior EEG slowing as an early marker of AD vulnerability [47,48]. Cortical and hippocampal atrophy are well-established pathological features of AD [49], which may underlie the altered EEG patterns observed in the parietal and temporal regions. Studies have demonstrated that theta frequency is among the earliest and most sensitive EEG biomarkers of AD pathology. Region-of-interest (ROI) analyses further revealed that the most critical brain areas—such as the parietal, temporal, and occipital lobes—are the most frequently associated with the progression of mild cognitive impairment (MCI) and AD [50]. The temporal and parietal regions are widely recognized as early targets of AD-related neuropathology, given their involvement in learning, memory, and sleep regulation [51]. While the general pattern of regional vulnerability is well-established, our study contributes to the field by demonstrating that task-related EEG features—collected from cognitively normal, middle-aged individuals—can capture subtle alterations in these regions before clinical symptoms appear. We believe that validating this neurophysiological vulnerability using an interpretable deep learning approach further supports the potential of EEG-based screening for preclinical AD risk in at-risk populations. Therefore, although the affected regions are expected, the non-invasive detection and classification of risk through explainable EEG patterns offers a novel and clinically relevant perspective.

We also investigated the misclassification patterns, which may be attributed either to incorrect sample labeling or non-neural artifacts such as eye movements or muscle-related noise. These findings emphasize the challenges of EEG-based classification in subclinical populations and highlight the need for robust denoising and signal quality control in future applications. The only fully reliable diagnosis of AD can be achieved through post-mortem neuropathological examination, which is generally unfeasible when collecting data for publicly available datasets [52]. As a result, there is an inherent risk of mislabeled samples, posing a critical challenge for real-world machine learning applications. In this study, the use of genetic risk status as a label may not fully reflect observable differences in EEG signals, potentially introducing label noise and limiting the discriminative power of the model. A promising approach is to incorporate model interpretability into the labeling process to mitigate sample labeling bias [35].

Overall, the proposed framework achieved consistent and interpretable classification performance in a challenging cross-subject setting. The results support the feasibility of using task-related EEG and deep neural networks for the early identification of AD genetic risk in cognitively normal individuals. Future work may explore model refinement through larger multi-site datasets, integration with other biomarkers (e.g., imaging or CSF), and prospective longitudinal validation. Despite the promising results and improved interpretability, several limitations should be acknowledged, which also provide directions for future research:

- Various mechanisms beyond genetic risk.While this study focuses on individuals stratified by APOE and PICALM genotypes, it is important to acknowledge that Alzheimer’s disease (AD) is a multifactorial condition, and genetic predisposition represents only one aspect of its etiology. Various biological mechanisms beyond genetic risk—such as neuroinflammation, metabolic dysfunction, and, notably, cholinergic deficits—may contribute to early AD-related brain changes, even in individuals without known genetic risk alleles. For example, individuals approaching the age of 60 may begin to experience early cholinergic dysfunction—an aspect of AD pathology [53]. Consequently, some misclassified subjects in the “N” (non-carrier) group may have exhibited preclinical AD driven by cholinergic deficits rather than genetic risk factors.
- Moderate classification performance. Although the model outperforms previous handcrafted feature methods [22], its ROC AUC remains around 60–61%, indicating only fair agreement. This reflects the challenge of identifying subtle EEG alterations in cognitively normal individuals. Future work should increase the sample size and diversity to enhance signal robustness and improve model generalizability.
- Lack of external validation. The model’s generalization was only tested on the A+P+ group, which was not used in training, and no independent external dataset was used. This limits the broader applicability of the results. Future studies should validate findings using external cohorts to assess reproducibility across sites and populations.
- Limited interpretability. Although post hoc visualization provided insights into the model’s decisions, the interpretations remain qualitative and lack biological confirmation. Future research should integrate EEG with multimodal imaging (e.g., MRI and PET) and explore explainability-guided training strategies to enhance the neuroscientific interpretability of learned features. While the interpretability analysis provides some visual explanations, there is still a lack of biological validation, making it unclear whether these features are truly associated with the pathological mechanisms of AD.

## 5. Conclusions

This study demonstrates the potential of EEG-based InterpretableCNN models for early Alzheimer’s disease (AD) risk prediction. The model achieved a maximum ROC AUC of 61.44% and a Cohen’s Kappa value of 0.23 under the STMT task condition, indicating fair classification agreement. The results show consistent performance across different task conditions, with slight variations in sensitivity metrics. When applied to the A+P+ group, the model exhibited reasonable generalizability, aligning with previous research on AD risk prediction. The model’s ability to focus on θ and α band activation in the parietal and temporal regions, along with frontal EEG channels, provides insights into brain biological activity changes related to genetic risk. This approach supports early AD risk screening, particularly in preclinical populations, and it offers a reference for future EEG-based risk assessments. Additionally, this study highlights the importance of using explainable models to enhance interpretability and trustworthiness, which are essential for clinical applications. The findings contribute to the growing body of AD prediction research and suggest that machine learning combined with neuroimaging can improve the understanding of AD’s neural mechanisms. Future studies with larger datasets and multimodal approaches could strengthen predictive models for broader clinical use and enhance targeted interventions for at-risk individuals.

In summary, this study demonstrates the potential of task-related EEG as a non-invasive biomarker for the early detection of preclinical Alzheimer’s disease. By leveraging explainable deep learning methods, we were able to identify frequency- and region-specific patterns that may reflect subtle neurophysiological changes prior to clinical onset. These findings have important clinical implications, as they support the development of EEG-based screening tools for at-risk populations, particularly middle-aged individuals with genetic susceptibility. Furthermore, the explainability of the model enhances its translational value, potentially facilitating clinical interpretation and decision-making. Future research should focus on validating these findings in larger, independent cohorts and exploring how such biomarkers can be integrated with other modalities (e.g., genetics and imaging) to improve diagnostic accuracy and personalized risk assessment.

## Figures and Tables

**Table 1:** Demographic characteristics of participants and trial counts in each group.

Groups	Subjects	Count	Age	Female/%	MSIT	STMT	All
N	1 2 6 7 8 10 13 14 15 16 17 18 19 22 23 24 25 26 28 29 31	21	55.29 ± 2.94	49.76	3176	2442	5618
A+P−	47 53 57 58 59 60 62 63 65 67 70 73 74 75 77 78 79 80	18	55.17 ± 3.03	51.45	2720	2151	4871
A+P+	32 34 35 37 39 40 41 42 44 48 52 54	12	56.67 ± 3.70	66.67	1738	1364	3102

**Table 2:** Detailed specifications of model.

Layer	Kernel	Filter	Group	Output Shape
Input				(N, 1, 60, 320)
Conv2D	(60, 1)	16	1	(N, 16, 1, 320)
Conv2D	(1, 64)	32	2	(N, 32, 1, 257)
ReLU				(N, 32, 1, 257)
BatchNorm2d				(N, 32, 1, 257)
AvgPool2d	(1, 257)			(N, 32)
Dense				(N, 2)
Softmaxt				(N, 2)

**Table 3:** Results across 100 folds of LPSO-CV (mean ± SD).

		ALL	MSIT	STMT
Validation	ROC AUC/%	60.84 ± 9.65	59.90 ± 8.79	61.44 ± 10.19
	Kappa/$\times 10^{-2}$	21.68 ± 19.29	19.80 ± 17.59	22.88 ± 20.37
	Sensitivity/%	49.34 ± 13.63	53.61 ± 14.40	48.96 ± 16.72
Test	Sensitivity/%	47.86 ± 8.75	51.05 ± 11.17	45.98 ± 13.58

## Data Availability

The preprocessed data of this study are available from the first author upon reasonable request at [2110108@stu.neu.edu.cn].

## Abbreviations

The following abbreviations are used in this manuscript:

AD	Alzheimer’s Disease
EEG	Electroencephalography
MSIT	Multi-Source Interference Task
STMT	Sternberg Memory Task
ROC AUC	Receiver Operating Characteristic Area Under the Curve
APOE	Apolipoprotein E
PICALM	Phosphatidylinositol Binding Clathrin Assembly Protein
LPSO-CV	Leave-p%-Subjects-Out Cross-Validation
InterpretableCNN	Interpretable Deep Learning Framework
MCI	Mild Cognitive Impairment
CAM	Class Activation Map
